# Supplementary material for: Effectiveness of formal onboarding for facilitating organizational socialization: A systematic review
Source: PLoS One. 2023 Feb 16;18(2):e0281823. doi: 10.1371/journal.pone.0281823 (PMC9934447; doi:10.1371/journal.pone.0281823)
Supplement: S1 Appendix — Appendix II: Studies excluded following full-text investigation with reason for exclusion. (DOCX) [file pone.0281823.s002.docx]

Appendix 1

Database: Web of Science Core Collection

Dates: April 21 2021 and November 9 2021

| Search # | Search terms |
| --- | --- |
| 1 | TS=((("new" or "newly" or "recent" or "recently") NEAR/1 (employ* or graduate* or hire* or professional* or qualified*) NEAR/1 ("adjustment" or intervention* or program* or sociali?ation*) ))  *Indexes=SCI-EXPANDED, SSCI, A&HCI, CPCI-S, CPCI-SSH, ESCI Timespan=All years* |
| 2 | TS=(newcomer* NEAR/1 ("adjustment" or sociali?ation*) )  *Indexes=SCI-EXPANDED, SSCI, A&HCI, CPCI-S, CPCI-SSH, ESCI Timespan=All years* |
| 3 | #2 OR #1  *Indexes=SCI-EXPANDED, SSCI, A&HCI, CPCI-S, CPCI-SSH, ESCI Timespan=All years* |
| 4 | TS=("apprentice" or aspirant$ or cadet$ or candidate$ or graduate$ or "intern" or  "interns" or neophyte$ or newcomer$ or novice$ or trainee$ or  "vocational learner$")  *Indexes=SCI-EXPANDED, SSCI, A&HCI, CPCI-S, CPCI-SSH, ESCI Timespan=All years* |
| 5 | TS=((("new" or "newly" or "recent" or "recently") NEAR/2 (employ* or hire* or professional* or “qualified”) ))  *Indexes=SCI-EXPANDED, SSCI, A&HCI, CPCI-S, CPCI-SSH, ESCI Timespan=All years* |
| 6 | TS=(("young*" or "junior*") NEAR/2 (employ* or hire* or professional*) )  *Indexes=SCI-EXPANDED, SSCI, A&HCI, CPCI-S, CPCI-SSH, ESCI Timespan=All years* |
| 7 | TS=("transition to practice" or "transition towards practice" or "transition into practice" or “school-to-work transition” or “early-career”)  *Indexes=SCI-EXPANDED, SSCI, A&HCI, CPCI-S, CPCI-SSH, ESCI Timespan=All years* |
| 8 | #7 OR #6 OR #5 OR #4  *Indexes=SCI-EXPANDED, SSCI, A&HCI, CPCI-S, CPCI-SSH, ESCI Timespan=All years* |
| 9 | TS=("apprenticeship$" or "coaching" or "graduate* fellowship$" or "internship$" or  "mentor*" or "onboarding" or "on-boarding" or "orientation" or "peer group$" or "preceptor$" or "residency" or  "traineeship$")  *Indexes=SCI-EXPANDED, SSCI, A&HCI, CPCI-S, CPCI-SSH, ESCI Timespan=All years* |
| 10 | TS=(((aspirant* or candidate* or "corporate trainee*" or graduate* or "graduate*  recruitment*" or "induction" or preceptor* or trainee* or "transition") NEAR/1  (program*) ))  *Indexes=SCI-EXPANDED, SSCI, A&HCI, CPCI-S, CPCI-SSH, ESCI Timespan=All years* |
| 11 | TS=(((“inservice” or postgraduate* or "vocational") NEAR/1 "training"))  *Indexes=SCI-EXPANDED, SSCI, A&HCI, CPCI-S, CPCI-SSH, ESCI Timespan=All years* |
| 12 | TS=((organi?ation* or professional*) NEAR/1 sociali?ation*)  *Indexes=SCI-EXPANDED, SSCI, A&HCI, CPCI-S, CPCI-SSH, ESCI Timespan=All years* |
| 13 | #12 OR #11 OR #10 OR #9  *Indexes=SCI-EXPANDED, SSCI, A&HCI, CPCI-S, CPCI-SSH, ESCI Timespan=All years* |
| 14 | #8 AND #13  *Indexes=SCI-EXPANDED, SSCI, A&HCI, CPCI-S, CPCI-SSH, ESCI Timespan=All years* |
| 15 | #3 OR #14  *Indexes=SCI-EXPANDED, SSCI, A&HCI, CPCI-S, CPCI-SSH, ESCI Timespan=All years* |
| 16 | TS=((cohort* or "case-control" or "cross-over" or " longitudinal" or observation* or "prospective" or "quasi-experimental" or random* or trial*) ) OR TS=((intervention* or program*) NEAR/2  (effect* or evaluat* or implement*) )  *Indexes=SCI-EXPANDED, SSCI, A&HCI, CPCI-S, CPCI-SSH, ESCI Timespan=All years* |
| 17 | #16 AND #15  *Indexes=SCI-EXPANDED, SSCI, A&HCI, CPCI-S, CPCI-SSH, ESCI Timespan=All years* |
| 18 | #16 AND #15  **Refined by:** PUBLICATION YEARS: ( 2021 OR 2008 OR 2020 OR 2007 OR 2019  OR 2006 OR 2018 OR 2017 OR 2016 OR 2015 OR 2014 OR 2013 OR 2012 OR 2011 OR 2010 OR 2009 )  *Indexes=SCI-EXPANDED, SSCI, A&HCI, CPCI-S, CPCI-SSH, ESCI Timespan=All years* |

Database: Scopus

Dates: April 21 2021 and November 9 2021

| Search # | Search terms |
| --- | --- |
| 1 | TITLE-ABS-KEY ( ( ( "new" OR "newly" OR "recent" OR "recently" ) W/1 ( employ* OR graduate* OR hire* OR professional* OR qualified* ) W/1 ( "adjustment" OR intervention* OR program* OR sociali?ation* ) ) ) |
| 2 | TITLE-ABS-KEY ( ( newcomer* W/1 ( "adjustment" OR sociali?ation* ) ) |
| 3 | #1 OR #2 |
| 4 | TITLE-ABS ( "apprentice" OR aspirant* OR cadet* OR candidate* OR graduate* OR "intern" OR "interns" OR neophyte* OR newcomer* OR novice* OR trainee* OR "vocational learner*" ) |
| 5 | TITLE-ABS ( ( "new" OR "newly" OR "recent" OR "recently" ) W/2 ( employ* OR hire* OR professional* OR "qualified" ) ) |
| 6 | TITLE-ABS ( ( "young*" OR "junior*" ) W/2 ( employ* OR hire* OR professional* ) ) |
| 7 | TITLE-ABS ( "transition to practice" OR "transition towards practice" OR "transition into practice" OR "school to work transition" OR "early-career" ) |
| 8 | #4 OR #5 OR #6 OR #7 |
| 9 | TITLE-ABS ( "apprenticeship*" OR "coaching" OR "graduate* fellowship*" OR "internship*" OR "mentor*" OR "onboarding" OR "on-boarding" OR "orientation" OR "peer group*" OR "preceptor*" OR "residency" OR "traineeship*" ) |
| 10 | TITLE-ABS ( ( aspirant* OR candidate* OR "corporate trainee*" OR graduate* OR "graduate* recruitment*" OR "induction" OR preceptor* OR trainee* OR "transition" ) W/1 ( program* ) ) |
| 11 | TITLE-ABS ( ( "inservice" OR postgraduate* OR "vocational" ) W/1 ( "training" ) ) |
| 12 | TITLE-ABS ( ( organi?ation* OR professional* ) W/1 ( sociali?ation* ) ) |
| 13 | #9 OR #10 OR #11 OR #12 |
| 14 | TITLE-ABS ( cohort* OR "case-control" OR "cross-over" OR " longitudinal" OR observation* OR "prospective" OR "quasi-experimental" OR random* OR trial* ) |
| 15 | TITLE-ABS ( ( intervention* OR program* ) W/2 ( effect* OR evaluat* OR implement* ) ) |
| 16 | #14 OR #15 |
| 17 | #8 AND #13 |
| 18 | #17 OR #3 |
| 19 | #18 AND #16 AND ( LIMIT-TO ( PUBYEAR , 2021 ) OR LIMIT-TO ( PUBYEAR , 2020 ) OR LIMIT-TO ( PUBYEAR , 2019 ) OR LIMIT-TO ( PUBYEAR , 2018 ) OR LIMIT-TO ( PUBYEAR , 2017 ) OR LIMIT-TO ( PUBYEAR , 2016 ) OR LIMIT-TO ( PUBYEAR , 2015 ) OR LIMIT-TO ( PUBYEAR , 2014 ) OR LIMIT-TO ( PUBYEAR , 2013 ) OR LIMIT-TO ( PUBYEAR , 2012 ) OR LIMIT-TO ( PUBYEAR , 2011 ) OR LIMIT-TO ( PUBYEAR , 2010 ) OR LIMIT-TO ( PUBYEAR , 2009 ) OR LIMIT-TO ( PUBYEAR , 2008 ) OR LIMIT-TO ( PUBYEAR , 2007 ) OR LIMIT-TO ( PUBYEAR , 2006 ) ) |

Appendix II

| **Paper** | **Reason for exclusion^1^** |
| --- | --- |
| Ahlborg, L., Hedman, L., Nisell, H., Felländer-Tsai, L., Enochsson, L. 2013. Simulator training and non-technical factors improve laparoscopic performance among OBGYN trainees. Acta Obstetricia et Gynecologica Scandinavica, 92, 10, 1194-1201 | 5 |
| Al-Dossary, R. N., Kitsantas, P., Maddox, P. J. 2016. Residency Programs and Clinical Leadership Skills Among New Saudi Graduate Nurses. Journal of Professional Nursing, 32, 2, 152-158. | 9 |
| Al-Dossary, R. N., Kitsantas, P., Maddox, P. J. 2016. Clinical decision-making among new graduate nurses attending residency programs in Saudi Arabia. Applied Nursing Research, 29, 25-30 | 9 |
| Allen, D. G., Shanock, L. R. 2013. Perceived organizational support and embeddedness as key mechanisms connecting socialization tactics to commitment and turnover among new employees. Journal of Organizational Behavior, 34, 3, 350-369 | 5 |
| Amoako, A. O., Pujalte, G. G. A., Kaushik, N., Riley, T. 2018. Patient Discomfort and Resident Confidence After Knee Intra-articular Injection Simulation Training: A Randomized Control Trial Study. Clinical Medicine Insights-Arthritis and Musculoskeletal Disorders, 11, 1-3 | 6 |
| au, K. E., Cheng, L. G., Pan, Z. Y., Gaillard, P. R., Hammer, L. 2011. Effect of a Preceptor Education Workshop: Part 1. Quantitative Results of a Hospital-Wide Study. Journal of Continuing Education in Nursing, 42, 3, 117-126 | 5 |
| Bagai, A., O'Brien, S., Al Lawati, H., Goyal, P., Ball, W., Grantcharov, T., Fam, N. 2012. Mentored simulation training improves procedural skills in cardiac catheterization: A randomized, controlled pilot study. Circulation: Cardiovascular Interventions, 5, 5, 672-679 | 6 |
| Banks, P., Roxburgh, M., Kane, H. Laider, W., Jones, M., Kydd, A., & Atkinson, J. 2011. Flying Start NHS: Easing the transition from student to registered health professional. Journal of Clinical Nursing, 20, 3567–3576. | 3 |
| Bansal, V. K., Raveendran, R., Misra, M. C., Bhattacharjee, H., Rajan, K., Krishna, A., Kumar, P., Kumar, S. 2014. A Prospective Randomized Controlled Blinded Study to Evaluate the Effect of Short-Term Focused Training Program in Laparoscopy on Operating Room Performance of Surgery Residents (CTRI /2012/11/003113). Journal of Surgical Education, 71, 1, 52-60 | 6 |
| Bastian, K. C., Marks, J. T. 2017. Connecting Teacher Preparation to Teacher Induction: Outcomes for Beginning Teachers in a University-Based Support Program in Low-Performing Schools. American Educational Research Journal, 54, 2, 360-394 | 6 |
| Bauer, T. N. Erdogan, B. Caughlin, D. Ellis, A. M. Kurkoski, J. 2020. Jump-Starting the Socialization Experience: The Longitudinal Role of Day 1 Newcomer Resources on Adjustment. Journal of Management, XX,, X, 1–36 | 6 |
| Bjerregaard, K. Haslam, S. A. Morton, T. 2016. How identification facilitates effective learning: the evaluation of generic versus localized professionalization training. International Journal of Training and Development, 20, 1, 17-37 | 6 |
| Bogetz, J. F., Gabhart, J. M., Rassbach, C. E., ers, L. M., Mendoza, F. S., Bergman, D. A., Blankenburg, R. L. 2015. Outcomes of a Randomized Controlled Educational Intervention to Train Pediatric Residents on Caring for Children With Special Health Care Needs. Clinical Pediatrics, 54, 7, 659-666 | 6 |
| Bonrath, E. M. Dedy, N. J. Gordon, L. E. Grantcharov, T. P. 2015. Annals of Surgery,262, 2, 205-212 | 5 |
| Boswell, W. R. Shipp, A. J. Payne, S. C. Culbertson, S. S. 2009. Changes in Newcomer Job Satisfaction Over Time: Examining the Pattern of Honeymoons and Hangovers Comprehensive surgical coaching enhances surgical skill in the operating room: A randomized controlled trial. Journal of Applied Psychology, 94, 4, 844-858 | 5 |
| Bühren, S. C., Nolte, C., Schneider, E. M., Kraußlach, H. 2019. Promoting health, participation and belonging of apprentices in work settings. Pravention und Gesundheitsforderung - Volume 14, Issue 1, 22-28 | 1 |
| Chan, G. K., Burns, E. M. 2021. Quantifying and Remediating the New Graduate Nurse Resident Academic-Practice Gap Using Online Patient Simulation. Journal of Continuing Education in Nursing, 52, 5, 240-247 | 3 |
| Chang, J. Chang, W. Jacobs, R. 2009. Relationship between participation in communities of practice and organizational socialization in the early careers of South Korean it employees. Human Resource Development International, 12, 4, 407-427 | 6 |
| Chapman, N. Oultram, S. 2008. Piloting E-Learning in a Radiation Oncology Department. Journal of Medical Imaging and Radiation Sciences, 39, 2, 81-85 | 4 |
| Claxton, R., Marks, S., Buranosky, R., Rosielle, D., Arnold, R. M. 2011. The educational impact of weekly E-mailed fast facts and concepts. Journal of Palliative Medicine, 14, 4, 475-481 | 9 |
| Cohen, E. R., Barsuk, J. H., Moazed, F., Caprio, T., Didwania, A., McGaghie, W. C., Wayne, D. B. 2013. Making July safer: Simulation-based mastery learning during intern boot camp. Academic Medicine, 88, 2, 233-239 | 8 |
| Coyle, C. E. Gleason, H. 2015. Personal care attendant (PCA) new hire orientation program: A statewide evaluation. Gerontologist, 55, 614-614 | 2 |
| Crimlisk, J. T., Gr, e, M. M., Krisciunas, G. P., Costello, K. V., Fern, es, E. G., Griffin, M. 2017. Nurse residency program designed for a large cohort of new graduate nurses: Implementation and outcomes. MEDSURG Nursing, 26, 2, 83-87 | 3 |
| Deetlefs, A. M. J., Chalmers, J., Tindall, K., Wiryakusuma-McLeod, C., Bennett, S., Hay, I., Humphries, J., Eady, M. J., Cronin, L., Rudd, K 2021. Applying behavioral insights to increase rural and remote internships: Results from two Randomized Controlled Trials. Journal of Behavioral and Experimental Economics, 92, XX. | 4 |
| Dotters-Katz, S. K., Chuang, A., Weil, A., Howell, J. O. 2018. Developing a pilot curriculum to foster humanism among graduate medical trainees. Journal of Education and Health Promotion, 7, 1, XX | 6 |
| Doughty, L., Sinnema, C., McKillop, A., Dixon, R. 2021. The impact of postgraduate education in transition to practice programmes on new graduate nurses' knowledge and skills: A pre- post survey design. Nurse Education Today, 102, XX-XX | 6 |
| Dufour, L., Escribano, P. I., Maoret, M. 2021. (How) Will I Socialize You? The Impact of Supervisor Initial Evaluations and Subsequent Support on the Socialization of Temporary Newcomers. Organization Science, 32, 3, 881-908 | 4 |
| Egan, T. M., Song, Z. 2008. Are facilitated mentoring programs beneficial? A randomized experimental field study. Journal of Vocational Behavior, 72, 3, 351-362 | 6 |
| El Akremi, A., Nasr, M. I., Richebé, N. 2014. Relational, organizational and individual antecedents of the socialization of new recruits. Management (France), 17, 5, 317-345 | 3 |
| Finn, K. M., Metlay, J. P., Chang, Y., Nagarur, A., Yang, S., Rigan, C. P., Iyasere, C. 2018. Effect of increased inpatient attending physician supervision on medical errors, patient safety, and resident education: A randomized clinical trial. JAMA Internal Medicine, 178, 7, 952-959 | 6 |
| Frank, K., Lombaard, H., Pattinson, R. C. 2009. Does completion of the Essential Steps in Managing Obstetric Emergencies (ESMOE) training package result in improved knowledge and skills in managing obstetric emergencies? South African Journal of Obstetrics and Gynaecology, 15, 3, 94-99 | 4 |
| Gaies, M. G., Morris, S. A., Hafler, J. P., Graham, D. A., Capraro, A. J., Zhou, J., Rigan, C. P., Ora, T. J. 2009. Reforming procedural skills training for pediatric residents: A randomized, interventional trial. Pediatrics, 124, 2, 610-619 | 8 |
| Garrison, E., Colin, S., Lemberger, O., Lugod, M. 2021. Interactive Learning for Nurses Through Gamification. The Journal of nursing administration, 51, 2, 95-100 | 6 |
| Ghazali, N. M., Hii, H. N., Wan Jaafar, W. M., Anuar, A., Aden, E., Yahyah, F. 2020. Significance of counselling orientation on supervision outcomes among trainee counsellors: Comparison between previous and current study in Malaysia. Journal of Critical Reviews, 7, 16, 222-232 | 3 |
| Gupta, D. K., Kh, ker, N., Stacy, K., Tatsuoka, C. M., Preston, D. C. 2017. Utility of combining a simulation-based method with a lecture-based method for fundoscopy training in neurology residency. JAMA Neurology, 74, 10, 1223-1227 | 6 |
| Halvorsen, F. H., Fosse, E., Mjål, O. 2011. Unsupervised virtual reality training may not increase laparoscopic suturing skills. Surgical Laparoscopy, Endoscopy and Percutaneous Techniques, 21, 6, 458-461 | 8 |
| Han, S., Obuch, J. C., Keswani, R. N., Hall, M., Patel, S. G. et al. 2020. Effect of individualized feedback on learning curves in EGD and colonoscopy: a cluster randomized controlled trial. Gastrointestinal Endoscopy, 91, 4, 882-893.e4 | 5 |
| Huang, X. L., Tsao, Y., Chung, H. C., Creedy, D. K. 2021. Effects of a mobile phone application for graduate nurses to improve central venous catheter care: A randomized controlled trial. Journal of Advanced Nursing. 77, 5, 2328-2339 | 8 |
| Jokisaari, M., & Nurmi, J. 2009. Change in newcomers’ supervisor support and socialization outcomes after organisational entry. Academy of Management Journal, 52, 527–544. | 3 |
| Justus, P. D., Appel, S. J. 2018. Simulation with Advanced Care Providers in a Nurse Residency Program. Journal for Nurses in Professional Development, 34, 4, 180-184 | 8 |
| Kamau, C. 2014. Outcomes of care programme approach, dual diagnosis, carer support and psychological therapy inductions. BJPsych Bulletin, 38, 4, 172-174 | 8 |
| Kammeyer-Mueller, J., Wanberg, C., Rubenstein, A., Song, Z. 2013. Support, undermining, and newcomer socialization: Fitting in during the first 90 days. Academy of Management Journal, 56, 4, 1104-1124 | 5 |
| Kessler, D., Pusic, M., Chang, T. P., Fein, D. M., Grossman, D. et al. 2015. Impact of just-in-time and just-in-place simulation on intern success with infant lumbar puncture. Pediatrics, 135, 5, e1237-e1246 | 8 |
| Kessler, D. O., Arteaga, G., Ching, K., Haubner, L., Kamdar, G. et al. 2013. Interns' Success With Clinical Procedures in Infants After Simulation Training. Pediatrics, 131, 3, E811-E820 | 8 |
| Kessler, D. O., Auerbach, M., Pusic, M., Tunik, M. G., Foltin, J. C. 2011. A randomized trial of simulation-based deliberate practice for infant lumbar puncture skills. Simulation in Healthcare, 6, 4, 197-203 | 6 |
| Knudsen, B. E., Matsumoto, E. D., Chew, B. H., Johnson, B., Margulis, V. 2006. A Randomized, Controlled, Prospective Study Validating the Acquisition of Percutaneous Renal Collecting System Access Skills Using a Computer Based Hybrid Virtual Reality Surgical Simulator: Phase I. Journal of Urology, 176, 5, 2173-2178. | 6 |
| Kotejoshyer, R., Gilmer, D. O., Namazi, S., Farr, D., Henning, R. A. 2021. Impact of a Total Worker Health(R) Mentoring Program in a Correctional Workforce. International Journal of Environmental Research and Public Health, 18, 16, XX-XX | 5 |
| Kowtha, N. R. 2009. Socialization tactics, newcomer work experience and information-seeking: An interactionist study. Academy of Management Annual Meeting Proceedings | 3 |
| Kozák, A., Krajcsák, Z. 2018. Retaining the rookie - Role clarification through mentorship. Human Systems Management, 37, 1, 95-103 | 3 |
| Lapointe, E., Vandenberghe, C., Boudrias, J-S. 2014. Organizational socialization tactics and newcomer adjustment: The mediating role of role clarity and affect‐based trust relationships. Journal of Occupational and Organizational Psychology, 87, 599–624 | 6 |
| Lau, K. W., Lee, P. Y., He, M. Y. 2018. 360 degree immersive videos: a way to improve organizational learning practices. Development and Learning in Organizations, 32, 6, 8-11 | 6 |
| Lauzier, M., Haccoun, R. R. 2014. The Interactive Effect of Modeling Strategies and Goal Orientations on Affective, Motivational, and Behavioral Training Outcomes. Performance Improvement Quarterly, 27, 2, 83-102 | 6 |
| Lee, G. I., Lee, M. R. 2018. Can a virtual reality surgical simulation training provide a self-driven and mentor-free skills learning? Investigation of the practical influence of the performance metrics from the virtual reality robotic surgery simulator on the skill learning and associated cognitive workloads. Surgical Endoscopy, 32, 1, 62-72 | 5 |
| Letourneau, R.M., McCurry, M.K. 2019. The Effect of Transition to Practice Programs on the Self-Assessment of Newly Licensed Registered Nurses' Confidence in Quality and Safety Competency Attainment. Nurs Educ Perspect. 40, 3, 151-156 | 3 |
| Liang, M. I., McCann, G. A., Rath, K. S., Backes, F. J., Cansino, C., Salani, R. 2014. Training the next generation of robotic surgeons using guided mentorship: A randomized controlled trial. Journal of Minimally Invasive Gynecology, 21, 6, 1075-1079 | 6 |
| Lindfors, K., Kaunonen, M., Huhtala, H., Paavilainen, E. 2021. Newly graduated nurses’ evaluation of the received orientation and their perceptions of the clinical environment: An intervention study. Scandinavian Journal of Caring Sciences, X, X, XX-XX | 8 |
| Liu, P. Q., Xu, S., Chen, Y., Yang, F., Si, Z. X. 2020. Perceived superior support and newcomer adjustment: A longitudinal study of new employees beginning their career. Journal of Chinese Human Resources Management, 11, 2, 1-18 | 5 |
| LoCasale-Crouch, J., Davis, E., Wiens, P., Pianta, R. 2012. The role of the mentor in supporting new teachers: Associations with self-efficacy, reflection, and quality. Mentoring & Tutoring: Partnership in Learning, 20, 3, 303-323 | 6 |
| Logishetty, K., Rudran, B., Cobb, J. P. 2019. Virtual reality training improves trainee performance in total hip arthroplasty: A randomized controlled trial. Bone and Joint Journal, 101, 12, 1585-1592 | 8 |
| Lonnbro, J., Nylen, K., Wallerstedt, S. M. 2019. Developing professional confidence in the art of prescribing-a randomized controlled study on structured collegial discussions during internship. European Journal of Clinical Pharmacology, 75, 5, 687-696 | 5 |
| Lowman, J. J. 2016. A Comparison of Three Professional Development Mechanisms for Improving the Quality of Standards-Based IEP Objectives. Communication Disorders Quarterly, 37, 4, 211-224 | 5 |
| Maschuw, K., Schlosser, K., Kupietz, E., Slater, E. P., Weyers, P., Hassan, I. 2011. Do soft skills predict surgical performance? A single-center randomized controlled trial evaluating predictors of skill acquisition in virtual reality laparoscopy. World Journal of Surgery, 35, 3, 480-486 | 8 |
| McCormick, E., Kerns, S. E. U., McPhillips, H., Wright, J., Christakis, D. A., Rivara, F. P. 2014. Training Pediatric Residents to Provide Parent Education: A Randomized Controlled Trial. Academic Pediatrics, 14, 4, 353-360 | 6 |
| McKenzie, D., Assaf, N., Cusolito, A. P. 2016. The demand for, and impact of, youth internships: evidence from a randomized experiment in Yemen. IZA Journal of Labor and Development, 5, 1 | 8 |
| McNatt, D. B., Judge, T. A. 2008. Self-efficacy intervention, job attitudes, and turnover: A field experiment with employees in role transition. Human Relations, 61, 6, 783-810 | 6 |
| Meng, W., Yue, P., Leung, J. W., Wang, H., Wang, X., Wang, F., et al. 2020. Impact of mechanical simulator practice on clinical ERCP performance by novice surgical trainees: A randomized controlled trial. Endoscopy, 52, 11, 1004-1013 | 5 |
| Meyer, G., Shatto, B., Delicath, T., von der Lancken, S. 2017. Effect of Curriculum Revision on Graduates' Transition to Practice. Nurse Educ. 42, 3, 127-132 | 3 |
| Miller, M., Mullen, A., Gardner, A. 2019. What clinical rotation factors have an impact on trainee success?. Clinical Teacher, 16, 3, 263-268 | 4 |
| Mitus, J. S. 2006. Organizational socialization from a content perspective and its effect on the affective commitment of newly hired rehabilitation counselors. Journal of Rehabilitation, 72, 2, 12-20 | 5 |
| Mizota, T., Kurashima, Y., Poudel, S., Watanabe, Y., Shichinohe, T., Hirano, S. 2018. Step-by-step training in basic laparoscopic skills using two-way web conferencing software for remote coaching: A multicenter randomized controlled study. American Journal of Surgery, 216, 1, 88-92 | 6 |
| Morris, L. L., Pfeifer, P., Catalano, R., Fortney, R., Nelson, G. et al. 2009. Outcome evaluation of a new model of critical care orientation. American Journal of Critical Care, 18, 3 | 6 |
| Murabit, A., Anzarut, A., Kasrai, L., Fisher, D., Wilkes, G. 2010. Teaching ear reconstruction using an alloplastic carving model. Journal of Craniofacial Surgery, 21, 6, 1719-1721 | 6 |
| Myers, J. S., Jaipaul, C. K., Kogan, J. R., Krekun, S., Bellini, L. M., Shea, J. A. 2006. Are discharge summaries teachable? The effects of a discharge summary curriculum on the quality of discharge summaries in an internal medicine residency program. Acad Med. 81, s5-8 | 8 |
| Nifadkar, S. S. 2020. Filling in the “Blank Slate”: Examining Newcomers’ Schemas of Supervisors During Organizational Socialization. Journal of Management, 46, 5, 666-693 | 3 |
| Nägele, C. Neuenschw, er, M. P. 2016. Apprentice-trainer relationship and work group integration in the first months of an apprenticeship. Empirical Research in Vocational Education and Training, 8, 1 | 3 |
| Olinover, M., Gidron, M., Yarmolovsky, J., Geva, R. 2021. Strategies for improving decision making of leaders with ADHD and without ADHD in combat military context. Leadership Quarterly, X, X, XX-XX | 3 |
| Otokiti, A., Ward, P., Dongol, M., Sideeg, A., Osman, M. et al. 2018. Implementation of a Peer-to-Peer Teaching Intervention Improved Electronic Discharge Summary Documentation by New Internal Medicine Residents. Medical Science Educator, 28, 2, 345-350 | 8 |
| Owings, C. R., Gaskins, S. W. 2020. Evaluation of a Community-Based Nurse Residency. Journal for Nurses in Professional Development, 36, 4, 185-190 | 3 |
| Parent, R. J., Plerhoples, T. A., Long, E. E., Zimmer, D. M., Teshome, M. et al. 2010. Early, Intermediate, and Late Effects of a Surgical Skills "Boot Camp" on an Objective Structured Assessment of Technical Skills: A Randomized Controlled Study. Journal of the American College of Surgeons, 210, 6, 984-989 | 8 |
| Perrot, S., Bauer, T. N., Abonneau, D., Campoy, E., Erdogan, B. et al. 2014. Organizational socialization tactics and newcomer adjustment: The moderating role of perceived organizational support. Group & Organization Management, 39, 3, 247-273 | 4 |
| Pillai, S., Manister, N. N., Coppolo, M. T., Ducey, M. S., McManus-Penzero, J. 2018. Evaluation of a Nurse Residency Program. Journal for Nurses in Professional Development, 34, 6, E23-E28 | 3 |
| Pimmer, C., Brühlmann, F., Odetola, T. D., Oluwasola, D. O., Dipeolu, O. et al. 2019. Facilitating professional mobile learning communities with instant messaging. Computers and Education, 128, 102-112 | 9 |
| Ploger, W., Scholl, D., Schule, C., Seifert, A. 2019. Development of trainee teachers' analytical competence in their induction phase - A longitudinal study comparing science and non-science teachers. Teaching and Teacher Education, Volume 85, 215-225 | 4 |
| Raub, S., Borzillo, S., Perretten, G., Schmitt, A. 2021. New employee orientation, role-related stressors and conflict at work: Consequences for work attitudes and performance of hospitality employees. International Journal of Hospitality Management, 94, XX-XX | 4 |
| Rausch, A., Seifried, J., Harteis, C. 2014. Missing effects of the professionalisation of in-house trainers within vocational education and training: Results from a longitudinal study. Zeitschrift fur Erziehungswissenschaft, 17, 127-147 | 1 |
| Redding, C., Smith, T. M. 2019. Supporting early career alternatively certified teachers: Evidence from the beginning teacher longitudinal survey. Teachers College Record, 121, 11, XX-XX. | 3 |
| Roche, J., Schoen, D., Kruzel, A. 2013. Human patient simulation versus written case studies for new graduate nurses in nursing orientation: A pilot study. Clinical Simulation in Nursing, 9, 6, e199-e205 | 8 |
| Rose, P. 2016. Interns proactively shaping their organizational experience: The mediating role of leader member exchange. Asia-Pacific Journal of Cooperative Education, 17, 3, 309-323 | 3 |
| Rossler, K. L., Hardin, K. 2020. Teaching Newly Licensed RNs to Build an Interprofessional Collaborative Practice. Journal of Continuing Education in Nursing, 51, 7, 331-337 | 7 |
| Proulx, D. M., Bourcier, B. J. 2008. Graduate nurses in the intensive care unit: an orientation model. Critical Care Nurse, 28, 4 | 3 |
| Rush, K. L., Adamack, M., Gordon, J., Janke, R., Ghement, I. R. 2015. Orientation and transition programme component predictors of new graduate workplace integration. Journal of Nursing Management, 23, 143–155 | 6 |
| Sampson, M., Melnyk, B. M., Hoying, J. 2019. Intervention Effects of the MINDBODYSTRONG Cognitive Behavioral Skills Building Program on Newly Licensed Registered Nurses' Mental Health, Healthy Lifestyle Behaviors, and Job Satisfaction. Journal of Nursing Administration, 49, 10, 487-495 | 8 |
| Sandau, K. E., Halm, M. A. 2010. Preceptor-based orientation programs: Effective for nurses and organizations? American Journal of Critical Care, 19, 2, 184-188 | 3 |
| Schout, B. M. A., Ananias, H. J. K., Bemelmans, B. L. H., D'Ancona, F. C. H., Muijtjens, A. M. M. et al. 2010. Transfer of cysto-urethroscopy skills from a virtual-reality simulator to the operating room: A randomized controlled trial. BJU International, 106, 2, 226-231 | 8 |
| Seimetz, J. A. 2021. External Evaluation of the Clinical Nurse Transition Program. Journal for Nurses in Professional Development, 37, 5, 278-284 | 3 |
| Seki, M., Otaki, J., Breugelmans, R., Komoda, T., Nagata-Kobayashi, S. et al. 2016. How do case presentation teaching methods affect learning outcomes? SNAPPS and the One-Minute preceptor. BMC Medical Education, 16, 1 | 8 |
| Shelesky, G., D'Amico, F., Marfatia, R., Munshi, A., Wilson, S. A. 2012. Does weekly direct observation and formal feedback improve intern patient care skills development? a randomized controlled trial. Family Medicine, 44, 7, 486-492 | 8 |
| Shrestha, R., Badyal, D., Shrestha, A. P., Shrestha, A. 2020. In-situ simulation-based module to train interns in resuscitation skills during cardiac arrest. Advances in Medical Education and Practice, 11, 271-285 | 4 |
| Siau, K., Hodson, J., Neville, P., Turner, J., Beale, A. et al. 2020. Impact of a simulation-based induction programme in gastroscopy on trainee outcomes and learning curves. World Journal of Gastrointestinal Endoscopy, 12, 3, 98-110 | 6 |
| Simon, L. S., Bauer, T. N., Erdogan, B., Shepherd, W. 2019. Built to last: Interactive effects of perceived overqualification and proactive personality on new employee adjustment. Personnel Psychology, 72, 213–240 | 5 |
| Sitzmann, T., Ely, K. 2010. Sometimes You Need a Reminder: The Effects of Prompting Self-Regulation on Regulatory Processes, Learning, and Attrition. Journal of Applied Psychology, 95, 1, 132-144 | 5 |
| Slate, K. A., Stavarski, D. H., Romig, B. J., Thacker, K. S. 2018. Longitudinal Study Transformed Onboarding Nurse Graduates. Journal for Nurses in Professional Development, 34, 2, 92-98 | 3 |
| Smith, L. G. E., Amiot, C. E., Smith, J. R., Callan, V. J., Terry, D. J. 2013. The Social Validation and Coping Model of Organizational Identity Development: A Longitudinal Test. Journal of Management, 39, 7, 1952-1978 | 5 |
| Smith, S., Lobo, V., Anderson, K. L., Gisondi, M. A., Sebok-Syer, S. S. 2021. A randomized controlled trial of simulation-based mastery learning to teach the extended focused assessment with sonography in trauma. Aem Education and Training, 5, 3, XX-XX | 8 |
| Solinger, O. N., van Olffen, W., Roe, R. A., Hofmans, J. 2013. On becoming (un)committed: A taxonomy and test of newcomer onboarding scenarios. Organization Science, 24, 6, 1640-1661 | 3 |
| Song, Y. F., Liu, Y. H., Shi, J. Q., Wang, M. 2017. Use of proactive socialization tactics and socialization outcomes: A latent growth model approach to understand newcomer socialization process. Academy of Management Discoveries, 3, 1, 42-63 | 3 |
| Soucek, R., Pospech, I., Moser, K. 2010. Promoting social competence of apprentices: Evaluation of a training intervention. Zeitschrift fur Arbeits- und Organisationspsychologie, 54, 4, 182-191 | 1 |
| Spagnoli, P., Caetano, A., Tanucci, G., De Sousa, V. L. 2012. Information-seeking behaviour: Implicit and explicit strategies during the organizational entry process. Management Research, 10, 1, 6-28 | 3 |
| Spector, N., Blegen, M. A., Silvestre, J., Barnsteiner, J., Lynn, M. R. et al. 2015. Transition to Practice in Nonhospital Settings. Journal of Nursing Regulation, 6, 1, 4-13 | 5 |
| St-Jean, E., Audet, J. 2013. The Effect of Mentor Intervention Style in Novice Entrepreneur Mentoring Relationships. Mentoring and Tutoring: Partnership in Learning, 21, 1, 96-119 | 5 |
| St-Jean, É, Tremblay, M. 2020. Mentoring for entrepreneurs: A boost or a crutch? Long-term effect of mentoring on self-efficacy. International Small Business Journal: Researching Entrepreneurship, 38, 5, 424-448 | 5 |
| Strong, H., Bissell, M. B., Collins, J., Hartery, A. 2021. Effectiveness of a 12-Month Emergency Radiology Curriculum for Improving Self-Confidence and Competence of Postgraduate Year 1 Radiology Residents-A Canadian Study. Canadian Association of Radiologists Journal-Journal De L Association Canadienne Des Radiologistes, 72, 4, 614-620 | 3 |
| Stone, M., O’Donnell, P., Williams, S. 2020. Preservice to in-service: impact of structured peer group supervision in the training of school psychology interns. Clinical Supervisor, 39, 1, 85-105 | 4 |
| Takeuchi, N., Takeuchi, T. 2009. A longitudinal investigation on the factors affecting newcomers' adjustment: Evidence from Japanese organizations. International Journal of Human Resource Management, 20, 4, 928-952 | 3 |
| Takeuchi, N., Takeuchi, T., Jung, Y. 2021. Making a successful transition to work: A fresh look at organizational support for young newcomers from an individual-driven career adjustment perspective. Journal of Vocational Behavior, 103587 | 8 |
| Tan, Y. L., Shen, X. J. 2016. Socialization content and adjustment outcome: A longitudinal study of Chinese employees beginning their career. Social Behavior and Personality, 44, 1, 161-176 | 3 |
| Tang, C., Liu, Y., Oh, H., Weitz, B. 2014. Socialization Tactics of New Retail Employees: A Pathway to Organizational Commitment. Journal of Retailing, 90, 1, 62-73 | 3 |
| Tews, M. J., Tracey, J. B. 2008. An empirical examination of posttraining on-the-job supplements for enhancing the effectiveness of interpersonal skills training. Personnel Psychology, 61, 2, 375-401 | 5 |
| Thammasitboon, S., Darby, J. B., Hair, A. B., Rose, K. M., Ward, M. A. et al. 2016. A theory-informed, process-oriented Resident Scholarship Program. Medical Education Online, 21, 1 | 3 |
| Tomprou, M., Nikolaou, I. 2013. Exploring the role of social influence in promise beliefs and information acquisition among newcomers. European Journal of Work and Organizational Psychology, 22, 4, 408-422 | 8 |
| Tsai, S. L., Chai, S. K., Hsieh, L. F., Lin, S., Taur, F. M. et al. 2008. The use of virtual reality computer simulation in learning Port-A cath injection. Advances in Health Sciences Education, 13, 1, 71-87 | 8 |
| Tseng, M. Y., Hwang, S. L. 2021. "SURVIVAL" intervention program effects on student nurses' transition to staff nurses: A quasi-experimental study. Nurse Education in Practice, 56, XX-XX | 4 |
| Ulrich, B., Ashlock, C. H., Krozek, C., Africa, L. M., Early, S. et al. 2010. Improving retention, confidence, and competence of new graduate nurses: Results from a 10-year longitudinal database. Nursing Economics, 28, 6, 363-376 | 3 |
| Uttley, C. M., Horm, D. M. 2008. Mentoring in early childhood professional development: Evaluation of the rhode island child development specialist apprenticeship program. Journal of Early Childhood Teacher Education, 29, 3, 237-252 | 3 |
| Von Websky, M. W., Raptis, D. A., Vitz, M., Rosenthal, R., Clavien, P. A. et al. 2013. Access to a simulator is not enough: The benefits of virtual reality training based on peer-group-derived benchmarks - A randomized controlled trial. World Journal of Surgery, 37, 11, 2534-2541 | 6 |
| Waterhouse, J., French, E., Puchala, N. 2014. The Impact of Socialisation on Graduates' Public Service Motivation - a Mixed Method Study. Australian Journal of Public Administration, 73, 2, 247-259 | 8 |
| West, J. A., Barnard, M. 2010. Increasing employee knowledge through online orientation. International Journal of Learning, 17, 7, 347-358 | 3 |
| Wylde, C. M., Mahrer, N. E., Meyer, R. M. L., Gold, J. I. 2017. Mindfulness for Novice Pediatric Nurses: Smartphone Application Versus Traditional Intervention. Journal of Pediatric Nursing-Nursing Care of Children & Families, Volume 36, 205-212 | 8 |
| Xu, F., Ma, L., Wang, Y., Yu, J., Li, D. et al. 2021. Effects of an Innovative Training Program for New Graduate Registered Nurses: A Comparison Study. SAGE Open, 11, 1 | 9 |
| Yang, C., Chen, A. 2020. The longitudinal empirical study of organizational socialization and knowledge sharing – from the perspective of job embeddedness. Interdisciplinary Journal of Information, Knowledge, and Management, 15, 1-23 | 8 |
| Zackoff, M.W., Lin, L., Israel, K., Ely, K., Raab, D., Saupe, J., Klein, M., Sitterding, M. 2020. The Future of Onboarding Implementation of Immersive Virtual Reality for Nursing Clinical Assessment Training. J Nurses Prof Dev, 36, 4, 235-240 | 3 |
| Zanchetta, M., Junker, S., Wolf, A. M., Traut-Mattausch, E. 2020. “Overcoming the Fear That Haunts Your Success” – The Effectiveness of Interventions for Reducing the Impostor Phenomenon. Frontiers in Psychology, 11 | 6 |
| Zhang, Y., Liao, J., Yan, Y., Guo, Y. 2014. Newcomers’ future work selves, perceived supervisor support, and proactive socialization in Chinese organizations. Social Behavior and Personality, 42, 9, 1457-1472 | 6 |
| Zhao, T., Lai, L., Fan, F., Li, H., Yao, X. 2020. Testing the efficacy and mechanisms of an authentic self-expression orientation in enhancing newcomer job performance: A longitudinal quasi-field experiment. Academy of Management Annual Meeting Proceedings, doi.org/10.5465/AMBPP.2020.173 | 2 |
| Zheng, D., Wu, H., Eisenberger, R., Shore, L. M., Tetrick, L. E. et al. 2016. Newcomer leader–member exchange: the contribution of anticipated organizational support. Journal of Occupational and Organizational Psychology, 89, 4, 834-855 | 3 |
| Zigmont, J. J., Wade, A., Edwards, T., Hayes, K., Mitchell, J. et al. 2015. Utilization of experiential learning, and the learning outcomes model reduces RN orientation time by more than 35%. Clinical Simulation in Nursing, 11, 2, 79-94 | 8 |
| Note 1: Papers were examined in relation to the inclusion criteria in the order they are presented in the list below. Noted in the table is the first unfulfilled criteria per paper.  1. Language – English  2. Publication status – Published or accepted for publication  3. Study design – Randomized controlled trials, quasi-randomized controlled trials, cohort studies, and case-control studies  4. Setting – Professional organizations internationally  5. Population – New professionals with a mean age of 18-30 years  6. Intervention – Any formal onboarding practice or program intended to facilitate the professional socialization of new professionals starting within the first three months of a new professional’s employment  7. Comparison – Informal onboarding practices or ‘treatment as usual’  8. Outcome – The extent to which new professionals are socialized. This is operationalized using the key adjustment indicators role clarity, task mastery, and social acceptance, as well as specific measures of socialization  9 Could not get in contact with author for missing details | |
