# Supplementary material for: Effectiveness of formal onboarding for facilitating organizational socialization: A systematic review
Source: PLoS One. 2023 Feb 16;18(2):e0281823. doi: 10.1371/journal.pone.0281823 (PMC9934447; doi:10.1371/journal.pone.0281823)
Supplement: Attachment — Submitted filename: Response to reviewers final.docx [file pone.0281823.s003.docx]

Response to reviewers PLOS ONE manuscript PONE-D-22-02533.

We wish to thank the reviewers for their careful reading of the manuscript and their valuable comments. Below you will find the comments listed in the left column. To the right you will find our responses. We are happy to make additional changes if the editors and reviewers do not find that these changes have sufficiently improved the manuscript.

Sincerely,

The authours

| Comments from Reviewer 1 | Authors response |
| --- | --- |
| I find the title a bit too long and confusing. A title should be catchy to attract readership. The summary title sounds better than the main title. | Thank you for this suggestion. We have changed the title as suggested. |
| Line 36- What is JBI? Write it in full first then abbreviate subsequently. See also line 37 for GRADE. Read through the document and improve on this. | Thank you for pointing this out. We have removed the acronyms in the abstract. |
| Abstract - The objectives should be actionable and well formulated. | Thank you for this comment. We believe that the objective is actionable. As this is the phrasing that we used in our preregistered study protocol, we would prefer not to change it. |
| Abstract - All tenses should be in past tense. | Thank you for recognizing this mistake. We have changed all tenses to past tense. |
| Line 65 is it “from” or “form”? | Thank you for acknowledging this mistake, it has been corrected. |
| Line 70-72 though complete but needs to be followed by another comment as to whether the goal is being fulfilled or not. As it stands, it is hanging. | Thank you for this comment. We have added a few words to the sentence for it to be more clearly connected to the others. |
| Line 80-83 is incomplete. | Thank you for pointing this out, we have added the reference in text, not just as a number in brackets. |
| Line 83 is also incomplete; the meta-analysis was by? | Thank you for acknowledging this mistake, we have completed the sentence. |
| In line 89, the authors have written in full the IWG model and followed by explaining what it is but failed to explain what the 4 Cs model stands for. Though not broadly discussed but it is important to explain what the 4 Cs are, do not assume that every reader knows. | Thank you for pointing this out, we have added the four Cs. We have not added an explanation of what the model suggests in more detail, in line with the fact that we have not presented the socialization tactics model in more detail. If the reviewer/editors think that we should expand on these models as well we would be happy to do so. |
| Objectives - Should the definition of terms be in the objectives section? | Thank you for this question. We believed that this was a good place to present the definitions we used, but we would be happy to move them elsewhere if you/the editors believe somewhere else would be better. |
| Objectives - The authors have the purpose in line 153 but not the objective, or are they one and the same thing? Remember the objectives give vision to your study so they should stand out and be clearly stated as much as possible. | Thank you. We have changed the word “purpose” to “objective” for clarity. |
| Objectives - What is PROSPERO? What about JBI? Write them in full first then abbreviate going forward. | Again, thank you for pointing out this mistake. We have added the full names to the text. |
| Objectives - Some of the definitions of terms are not clear eg ‘Treatment as usual’. Authors should give the definition as used in the study and how it was measure/ applied. | Thank you for this comment. We believe that we have provided a clear definition of “treatment as usual”. We would be happy to add further specific details that the reviewer believes would improve the manuscript. |
| Inclusion Criteria - The authors have talked about those who were excluded, so who were included given that in this section you are talking about the inclusion criteria. There is a mix-up on the exclusion criteria, so either the authors change the sub-title to read ‘exclusion and inclusion criteria’ or discuss more the inclusion criteria as guided by the subtitle. | Thank you, this was a valuable comment. We have added “exclusion” to the title and text (in response to a comment by reviewer 2 we have also moved this section to the Methods). |
| Data extraction - The section has a lot of repetition revise to minimize on repetition and make the section clearer. | We are grateful for this comment. We have added a few words that we believe change the overall impression. We have also removed one “data” to reduce the number of repetitions. |
| Data extraction - Exactly how was data extracted? This is not clear. The authors need to give a blue print of the process, this has not come out clearly in the explanation given. | Thank you. We have added a few words regarding the extractions process. We find the text informative enough as is but would gladly add additional details if the reviewer may help us by suggesting what is missing. |
| Data synthesis and assessment of certainty in the findings - It is important to explain further how GRADE ratings were used. This is because the purpose of the methodology section is to give a step-by-step process to make it as clear as possible so that somebody else can use it to carry out a similar study elsewhere. | We appreciate this comment and have added an explanation of how the GRADE rating is performed. |
| Results - Line 287 & 288 - Why should authors follow a subtitle with another subtitle without saying anything under the results subsection to necessitate another subtitle. | Thank you, this was a good comment. We have accepted the proposal (in the following comment) to remove the “Study inclusion” subtitle. |
| Study inclusion- This subtitle is a bit confusing and the reader might think that the authors are talking about the inclusion criteria as above when it is not the case. The authors might want to change to a relevant sub-title or simply leave the section directly under the results sub heading. | Thank you for this suggestion. We have changed the text accordingly. |
| Methodological Quality - Stick to past tense. | Thank you, we have changed to past tense. |
| Findings - This section is clear with the process being clearly discussed in detail. There is however very little on findings. More details on the findings should be presented and the tables interpreted. There is also need to state the implications of the findings and relate it to the objectives of the study. This will bring clarity to the findings. | Thank you for this comment. We have added details about the specific programs and/or practices that were/were not effective in supporting newcomers’ professional adjustment, included a summarizing comment/interpretation, and related the results to the objectives. |
| Discussion - This section is well done and it discusses clearly both practices and programs in line with the purpose of the study. There is need in the discussion however to mention the importance of this approach to knowledge mining as compared to mining of primary data. | Thank you. We are not sure if we have correctly understood this comment, but we have tried at including a comment at the end of our Limitations section that points at the value of conducting systematic reviews to build knowledge. |
| Line 531- is it founding or funding? | Thank you for pointing this out. We have corrected our mistake. |
| References are well done but need but with very many typographical errors. The authors should be keen on referencing protocols | Thank you. We have formatted the references according to the guidelines. |

| Comments from Reviewer 2 | Response from authors |
| --- | --- |
| In the abstract, I would recommend to include the ‘inclusion criteria’ within the ‘methods’ section rather than standing separately. | Thank you, this was a valuable comment. We have moved the inclusion criteria to the methods section. |
| Introduction - I forward the same recommendation regarding ‘inclusion criteria’ in the main body of the manuscript. | Thank you. We have moved the inclusion and exclusion criteria to the methods section. |
| In addition, it would make more sense if the objective section does not stand alone but comes as a wrapping up of the introduction section that drew heavily on the critical review of the relevant literature. | Thanks, this was another valuable comment. We have integrated the objectives section with the introduction. |
| Results - The results section could have been enriched with presentation of findings on the proposed links between onboarding practices and programs, on the one hand, and role clarity, task mastery, and social acceptance, on the other hand. The studies must have included explanatory hypotheses on how the associations between the independent and outcome variables come about. | Thank you for this comment. We have added details about the specific programs and/or practices that were/were not effective in supporting newcomers’ professional adjustment, included a summarizing comment/interpretation, and related the results to the objectives. We refrain from talking in terms of “mechanisms of change” or “explanatory hypotheses” as the authors of the included papers unfortunately did not include such information explicitly. |
| Conclusions and recommendations - …Besides, the figure that describes the screening process, the reasons for the exclusion of some of the studies were not clearly stated. It would help if that information was provided as the authors did for the rest of the methodological issues. | We are grateful for this comment and have added a clarification in the text. We are however not sure if this is the kind of clarification that the reviewer was looking for, and would be happy to make further changes/clarifications. |
